# A Subadult Specimen of *Rubeosaurus ovatus* (Dinosauria: Ceratopsidae), with Observations on Other Ceratopsids from the Two Medicine Formation

**DOI:** 10.1371/journal.pone.0022710

**Published:** 2011-08-10

**Authors:** Andrew T. McDonald

**Affiliations:** Department of Earth and Environmental Science, University of Pennsylvania, Philadelphia, Pennsylvania, United States of America; Raymond M. Alf Museum of Paleontology, United States of America

## Abstract

**Background:**

Centrosaurine ceratopsids are well known from the middle Campanian Upper Two Medicine Formation of Montana. Four taxa have been named: *Brachyceratops montanensis*, *Rubeosaurus ovatus*, *Einiosaurus procurvicornis*, and *Achelousaurus horneri*. *Rubeosaurus* has been historically the most enigmatic of these taxa; only two specimens, the holotype caudal parietal bar and a referred incomplete skull, have been assigned to *Rubeosaurus*.

**Methodology/Principal Findings:**

A revised interpretation of the parietal processes of USNM 14765, the partial skeleton of a subadult centrosaurine formerly referred to *Brachyceratops*, indicates that it shares a P5 spike with the holotype of *Rubeosaurus ovatus* and should therefore be referred to that taxon. *Brachyceratops* is considered a *nomen dubium*.

**Conclusions/Significance:**

USNM 14765 provides additional anatomical information for *Rubeosaurus ovatus*. These new data are incorporated into a recent phylogenetic analysis of centrosaurine relationships; *Rubeosaurus* appears as the sister taxon of a clade composed of *Einiosaurus*, *Achelousaurus*, and *Pachyrhinosaurus*.

## Introduction

Centrosaurine ceratopsids are among the most ornate dinosaurs, sporting all manner of spikes, hooks, and protuberances on their parietosquamosal frills. Distinguished primarily by the morphologies of their cranial ornamentation, centrosaurines include *Diabloceratops eatoni* [1], *Albertaceratops nesmoi* [2], *Avaceratops lammersi* [3], [4], *Sinoceratops zhuchengensis* [5], an unnamed centrosaurine from the Belly River Group [6], *Centrosaurus brinkmani* [7], *Centrosaurus apertus* [8], [9], *Styracosaurus albertensis* [10], [11], *Rubeosaurus ovatus* [12], [13], *Einiosaurus procurvicornis* [14], *Achelousaurus horneri* [14], *Pachyrhinosaurus canadensis* [15], [16], and *Pachyrhinosaurus lakustai* [17]. Many centrosaurines, such as *Centrosaurus*, *Styracosaurus*, *Einiosaurus*, and *Pachyrhinosaurus*, are known from multiple skulls and skeletons or bone bed material. Others, such as *Diabloceratops*, *Albertaceratops*, *Sinoceratops*, and *Achelousaurus*, are known from isolated but well preserved specimens. In contrast, *Rubeosaurus* is based upon highly incomplete material and historically has been among the most mysterious ceratopsids.

**Figure 1:**
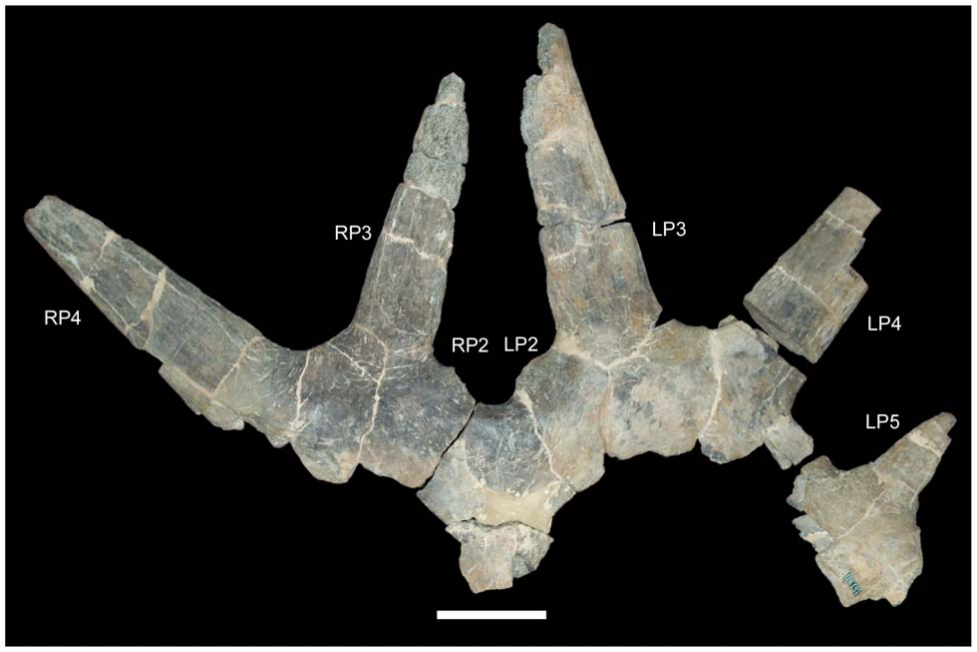
USNM 11869, holotype of *Rubeosaurus ovatus*. Caudal parietal bar in dorsal view. *Abbreviations*: *LP2*, left P2 process; *LP3*, left P3 process; *LP4*, left P4 process; *LP5*, left P5 process; *RP2*, right P2 process; *RP3*, right P3 process; *RP4*, right P4 process. Scale bar equals 10 cm. Copyright Smithsonian Institution, all rights reserved.

**Figure 2:**
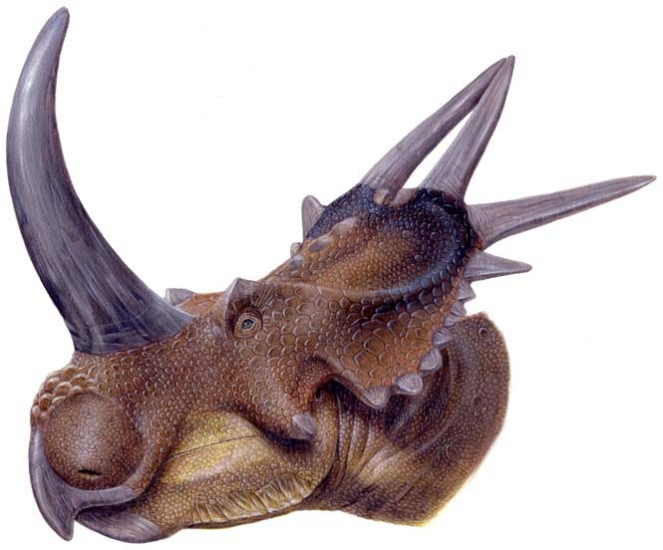
Life restoration of *Rubeosaurus ovatus*. Nasal and postorbital ornamentation based upon MOR 492, parietal ornamentation based upon USNM 11869. Artwork by Lukas Panzarin. This is the color version of the restoration that appeared in McDonald and Horner [13].

*Rubeosaurus ovatus* was originally named as a new species of *Styracosaurus* by Gilmore [12] based upon the caudal parietal bar of a large centrosaurine from the Upper Two Medicine Formation of Montana (Fig. 1) [13], [14]. This specimen, USNM 11869, exhibits an unusual feature: medially inclined P3 spikes. Even with the flurry of new centrosaurines discovered in the last two decades, this morphology remains unique to USNM 11869 and therefore can be considered an autapomorphy of *Rubeosaurus ovatus*. McDonald and Horner [13] described an incomplete centrosaurine skull, MOR 492, from approximately the same stratigraphic level as USNM 11869; MOR 492 was referred to *R. ovatus* due to the inferred medial inclination of the larger of the two preserved parietal spikes, which was interpreted as a P3. The referral of MOR 492 added considerably to the known anatomy of *R. ovatus*, especially concerning the nasal and postorbital ornamentation, and encouraged the first life restoration of the species (Fig. 2). A phylogenetic analysis suggested that *R. ovatus* was not congeneric with *Styracosaurus albertensis*, but instead closely related to *Einiosaurus procurvicornis*, prompting the erection of the new genus *Rubeosaurus* [13].

Recent examination of USNM 14765, a nearly complete but disarticulated centrosaurine skull with a partial postcranium described by Gilmore in 1939 [18], revealed features shared with USNM 11869, the holotype of *Rubeosaurus ovatus*. Thus, USNM 14765 is referred herein to *R. ovatus* and is the first immature individual of this species to be recognized. The preserved skull elements of USNM 14765 supplement USNM 11869 and MOR 492 and provide additional information on the skull of *Rubeosaurus*. These new data are incorporated into the most recent phylogenetic analysis of centrosaurine relationships [6]. The reassessment of USNM 14765 also encouraged a fresh evaluation of MOR 492. In addition to the three specimens of *Rubeosaurus ovatus*, several fragmentary but intriguing ceratopsid specimens from the Upper Two Medicine Formation were considered.

Institutional Abbreviations: MOR, Museum of the Rockies, Bozeman, Montana, USA; USNM, National Museum of Natural History, Washington, D.C., USA.

## Results and Discussion

### 1. *Rubeosaurus ovatus*

**Systematic Paleontology.**

Dinosauria Owen, 1842 [19]

Ornithischia Seeley, 1887 [20]

Ceratopsia Marsh, 1890 [21]

Ceratopsidae Marsh, 1888 [22]

Centrosaurinae Lambe, 1915 [23]

*Rubeosaurus* McDonald and Horner, 2010 [13]

*Rubeosaurus ovatus* Gilmore, 1930 [12]

**Synonymies.**

1930 *Styracosaurus ovatus* Gilmore, p. 36

2010 *Rubeosaurus ovatus* McDonald and Horner, p. 157

#### Holotype

USNM 11869, caudal parietal bar.

#### Referred Material

MOR 492, incomplete skull including partial fused left and right nasals (the left and right premaxillary processes of the nasals are also present; these have been broken off at their bases (fig. 1B, C in [13]), partial left premaxilla, partial left postorbital, proximal portion of median parietal bar, and right lateral parietal bar. USNM 14765, partial skull and postcranium including rostral, partial left premaxilla, a fragment of the nasal horncore, partial right maxilla, left circumorbital region (lacrimal, palpebral, prefrontal, postorbital, and jugal), supraoccipital, partial parietal, a dorsal vertebra, a dorsal rib, left scapula, right and left femora, and two phalanges.

#### Specific Diagnosis (as for genus by monotypy; modified from McDonald and Horner [13])

Centrosaurine ceratopsid diagnosed by a single autapomorphy: medially inclined P3 spikes. Also distinguished by the following unique combination of characters: elongate, tapering nasal horncore as in *Sinoceratops zhuchengensis*, *Centrosaurus brinkmani*, *Centrosaurus apertus*, *Styracosaurus albertensis*, and *Einiosaurus procurvicornis*; nasal horncore erect as in *Sinoceratops zhuchengensis*, *Centrosaurus brinkmani*, *Centrosaurus apertus*, and *Styracosaurus albertensis*; short, dorsally-projecting postorbital horncore with rounded apex as in unmodified adult specimens of *Styracosaurus albertensis* and *Einiosaurus procurvicornis*; P3 spike as in adult specimens of *Styracosaurus albertensis*, *Einiosaurus procurvicornis*, *Achelousaurus horneri*, *Pachyrhinosaurus canadensis*, and *Pachyrhinosaurus lakustai*; P3 spike is straight as in *Einiosaurus procurvicornis*; P4 spike as in adult specimens of *Styracosaurus albertensis*; tapering P5 spike shorter than the P3 and P4 as in adult specimens of *Styracosaurus albertensis*.

#### Locality and Horizon

All specimens were found in the vicinity of Landslide Butte on the Blackfeet Nation, Glacier County, Montana. The exact localities of USNM 11869 and USNM 14765 are unknown. Precise locality data for MOR 492 are on file at the Museum of the Rockies. USNM 11869 and MOR 492 were both collected in the Upper Two Medicine Formation, approximately 60 meters below the contact with the overlying Bearpaw Formation [13], in rocks dating to 75-74 Ma (middle Campanian) [24], [25]. According to Gilmore (p. 12 in [18]), USNM 14765 was collected about one mile from the type quarry of *Brachyceratops* at “approximately the same level in the formation”. If accurate, this means that USNM 14765 was also collected approximately 60 meters below the Two Medicine-Bearpaw contact. Specimens of *Einiosaurus procurvicornis* are known from approximately 45 meters below the contact, while those of *Achelousaurus horneri* came from approximately 20 meters below the contact [14].

#### Note on *Brachyceratops*

Before elaborating further on the new findings concerning *Rubeosaurus*, it is necessary to thoroughly address *Brachyceratops montanensis*, the first centrosaurine named from the Two Medicine Formation [26], [27]. *Brachyceratops* was named by Gilmore in 1914 [26] based upon the remains of five individuals from the same quarry. The type quarry of *Brachyceratops* is approximately 60 meters below the contact between the Two Medicine and Bearpaw formations [14], at the same stratigraphic level as USNM 11869 and MOR 492, the holotype and referred specimens of *Rubeosaurus* [13]. Thus, the taxonomic decision that immediately arises is whether to consider *Rubeosaurus ovatus* a junior synonym of *Brachyceratops montanensis*. This possibility was also raised by Dodson [28].

**Figure 3:**
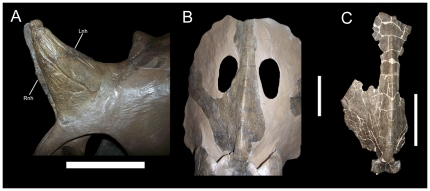
Morphology of *Brachyceratops montanensis*. Unfused right and left halves of the nasal horncore of USNM 7951 (holotype) in (A) left lateral view. Parietal of USNM 7951 (holotype) in (B) dorsal view. Parietal of USNM 7950 in (C) dorsal view. *Abbreviations*: *Lnh*, left half of nasal horncore; *Rnh*, right half of nasal horncore. Scale bars equal 10 cm. Copyright Smithsonian Institution, all rights reserved.

As thoroughly explicated by Sampson et al. [29], the holotype (USNM 7951) and other specimens from the type quarry of *Brachyceratops* are juvenile centrosaurines. USNM 7951 exhibits an unfused nasal horncore, a feature concordant with juvenile status (Fig. 3A). Furthermore, the two well preserved partial parietals from the *Brachyceratops* quarry (USNM 7951 and 7950; Fig. 3B, C) are missing most of their caudal bars and do not display features that could be classified as incipient versions of the diagnostic epiparietal morphologies in the frills of adult *Rubeosaurus*, *Einiosaurus*, or *Achelousaurus*. Finally, no elements from the *Brachyceratops* quarry exhibit autapomorphies or a unique combination of characters by which the taxon could be diagnosed. *Brachyceratops montanensis* should therefore be considered a *nomen dubium*.

Gilmore [18] referred USNM 14765 to *Brachyceratops* as a putative adult, an assignment upheld by Dodson [28]. However, the current study concurs with Sampson et al. [29] that USNM 14765 is a subadult. The parietal of USNM 14765 exhibits less extensive long-grained bone texture than the parietals of *Brachyceratops*, though a combination of long-grained and mottled bone texture is still present [29], suggesting that USNM 14765 is an immature centrosaurine [30]; Brown et al. [31] assigned USNM 14765 to their stage D. Moreover, USNM 14765 does not share any features exclusively with specimens from the *Brachyceratops* quarry.

**Figure 4:**
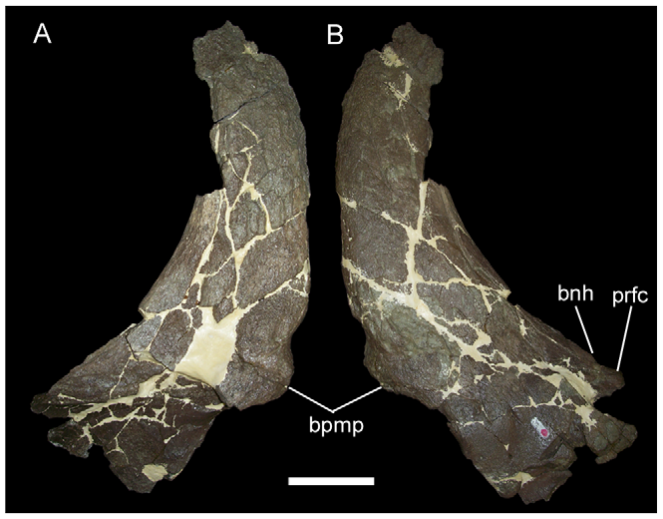
MOR 492, nasals of *Rubeosaurus ovatus*. Fused left and right nasals in (A) right lateral and (B) left lateral views. *Abbreviations*: *bnh*, base of nasal horncore; *bpmp*, base of premaxillary process; *prfc*, prefrontal contact. Scale bar equals 10 cm.

**Figure 5:**
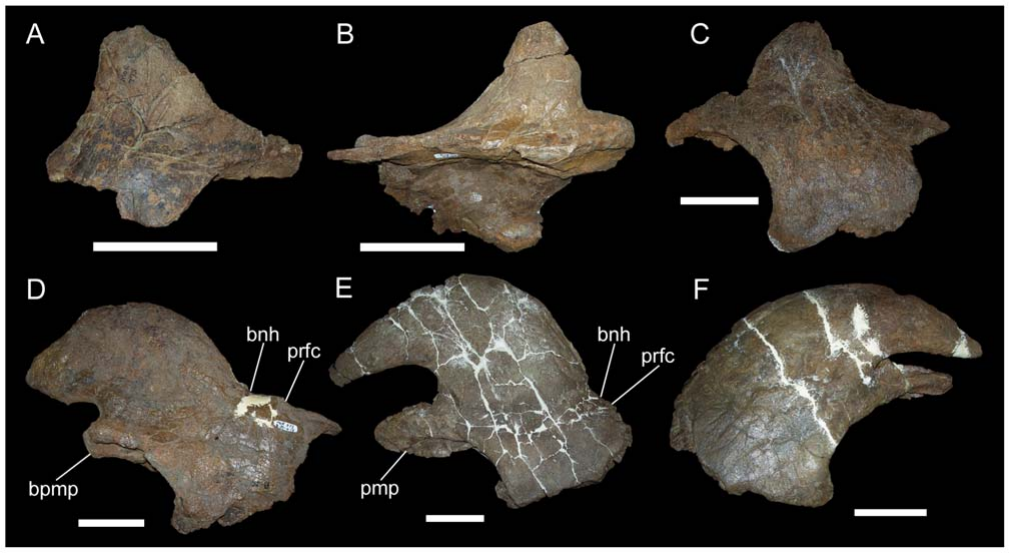
Nasals of *Einiosaurus procurvicornis*. (A) MOR 373-DR-85, unfused right nasal in lateral view; (B) MOR 373-8-3-87-9, fused left and right nasals in right lateral view; (C) MOR 373-7-6-86-9, fused left and right nasals in left lateral view; (D) MOR 373-8-20-6-14, fused left and right nasals in left lateral view; (E) MOR 456-8-9-6-1 (holotype), fused left and right nasals in left lateral view; (F) MOR 456-8-13-7-5, fused left and right nasals in right lateral view. *Abbreviations*: *bnh*, base of nasal horncore; *bpmp*, base of premaxillary process; *pmp*, premaxillary process; *prfc*, prefrontal contact. Scale bars equal 10 cm.

**Figure 6:**
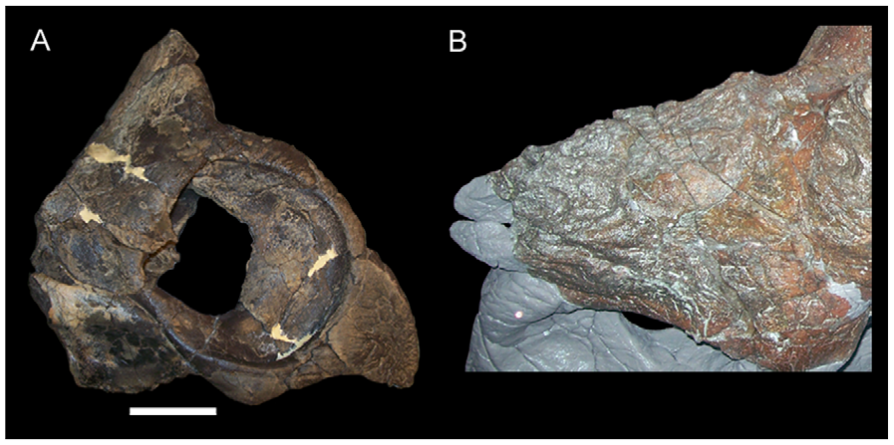
Nasals of *Achelousaurus horneri*. (A) MOR 591, articulated rostral, premaxillae, nasals, and rostral portions of the maxillae in right lateral view; (B) MOR 485 (holotype), nasals in left dorsolateral view. Scale bar in A equals 10 cm. MOR 485 was photographed behind glass, so a scale bar could not be applied; for scale, see Sampson (fig. 3 in [14]).

The erect, slightly recurved nasal horncore of MOR 492 (Fig. 4) does resemble that of USNM 7951; however, it is apparent from other centrosaurines that such horncores in immature individuals, such as USNM 7951, could potentially develop into a variety of adult morphologies and not necessarily into the tall, recurved horncore of MOR 492. The recurved nasal horncores of juvenile and subadult *Einiosaurus procurvicornis* (Fig. 5A–C) grew into the strongly procurved horncores of adults (Fig. 5D–F) [14], [29], while those of subadult *Achelousaurus horneri* (Fig. 6A) grew into pachyostotic bosses restricted to the nasals (Fig. 6B) [14], [29]. In perhaps the most extreme example, the nasal horncores of juvenile and subadult *Pachyrhinosaurus lakustai* developed into massive pachyostotic bosses that extend onto the prefrontals [17]. To assign MOR 492 to the same taxon as USNM 7951 because of gross similarity in the shapes of their nasal horncores would be to ignore the ontogenetic changes in those other centrosaurines. The similarity of juveniles across centrosaurine species and lack of diagnostic features in the material of *Brachyceratops* mean that there is simply too much uncertainty regarding the morphology of *Brachyceratops* to assign additional specimens (i.e. USNM 11869, USNM 14765, and MOR 492) to the taxon.

#### Features of *Rubeosaurus ovatus*: USNM 11869, USNM 14765, and MOR 492

**Figure 7:**
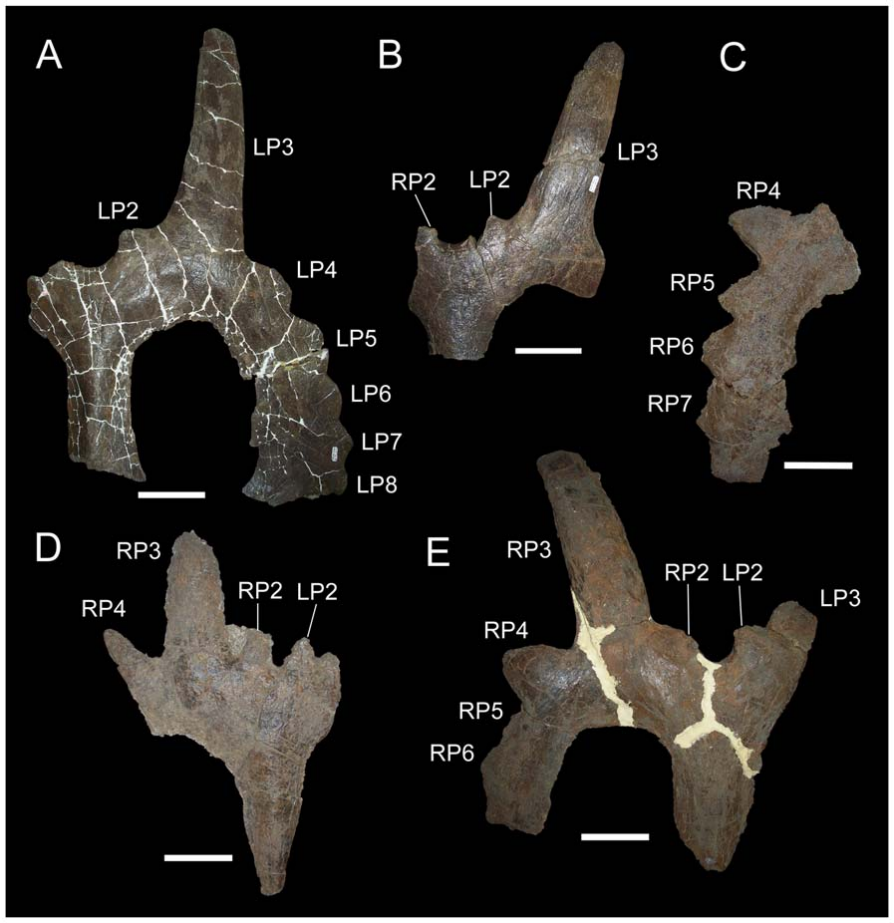
Parietals of *Einiosaurus procurvicornis*. (A) MOR 456-8-9-6-1 (holotype), parietal in dorsal view; (B) MOR 456-8-27-87-2, parietal in dorsal view; (C) MOR 373-001, right lateral parietal bar in dorsal view; (D) MOR 373-6-28-6-4, parietal in dorsal view; (E) MOR 373-7-9-87, parietal in dorsal view. *Abbreviations*: *LP2*, left P2 process; *LP3*, left P3 process; *LP4*, left P4 process; *LP5*, left P5 process; *LP6*, left P6 process; *LP7*, left P7 process; *LP8*, left P8 process; *RP2*, right P2 process; *RP3*, right P3 process; *RP4*, right P4 process; *RP5*, right P5 process; *RP6*, right P6 process; *RP7*, right P7 process. Scale bars equal 10 cm.

**Figure 8:**
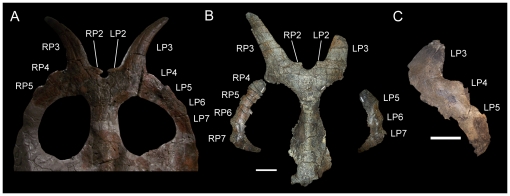
Parietals of *Achelousaurus horneri*. (A) MOR 485 (holotype), parietal in dorsal view; (B) MOR 571, parietal in dorsal view; (C) MOR 591, left lateral parietal bar in dorsal view. *Abbreviations*: *LP2*, left P2 process; *LP3*, left P3 process; *LP4*, left P4 process; *LP5*, left P5 process; *LP6*, left P6 process; *LP7*, left P7 process; *RP2*, right P2 process; *RP3*, right P3 process; *RP4*, right P4 process; *RP5*, right P5 process; *RP6*, right P6 process; *RP7*, right P7 process. Scale bars in B and C equal 10 cm. MOR 485 was photographed behind glass, so a scale bar could not be applied; for scale, see Sampson (fig. 3 in [14]).

The holotype of *Rubeosaurus ovatus*, USNM 11869, exhibits the sole autapomorphy of the taxon (medially-inclined P3 spikes). It also possesses elongate, laterally-inclined P4 spikes and a laterally-projecting left P5 spike that is considerably shorter than the left and right P3 and P4 spikes (Fig. 1). The medial-most epiparietals, the left and right P2 loci, are small, rounded, medially-inclined, and dorsoventrally compressed as in *Einiosaurus procurvicornis* (Fig. 7A, B, D, E), *Achelousaurus horneri* (Fig. 8A, B), and some specimens of *Styracosaurus albertensis* [11]. In contrast to *Centrosaurus* spp. [7], [9] and *Styracosaurus* [11], the P1 locus is absent as in *Einiosaurus* (Fig. 7A, B, D, E) [14], *Achelousaurus* (Fig. 8A, B) [14], and *Pachyrhinosaurus* spp. [16], [17].

**Figure 9:**
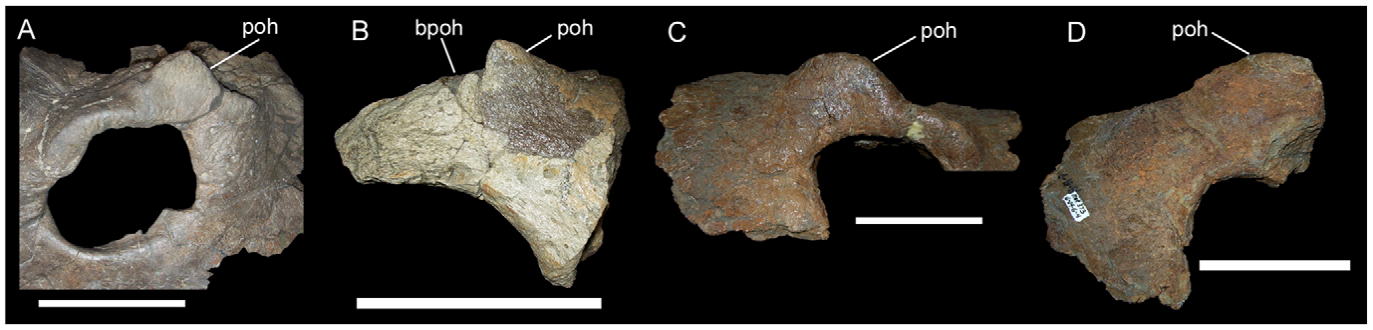
Postorbitals of *Rubeosaurus ovatus* (A and B) and *Einiosaurus procurvicornis* (C and D). (A) USNM 14765, articulated left jugal, lacrimal, palpebral, prefrontal, and postorbital in lateral view (Copyright Smithsonian Institution, all rights reserved); (B) MOR 492, partial left postorbital in lateral view; (C) MOR 373-6-26-6-3, articulated right prefrontal, palpebral, and postorbital in lateral view; (D) MOR 373-6-24-6-4, right postorbital in lateral view. *Abbreviations*: *bpoh*, base of postorbital horncore; *poh*, postorbital horncore. Scale bars equal 10 cm.

As explained above, USNM 14765 is very likely a subadult centrosaurine. The postorbital of USNM 14765 exhibits a low, rounded horncore (Fig. 9A), similar to that of MOR 492 (Fig. 9B) [13] and to those of subadult and unmodified adult *Styracosaurus albertensis* [11] and *Einiosaurus procurvicornis* (Fig. 9C, D) [14].

**Figure 10:**
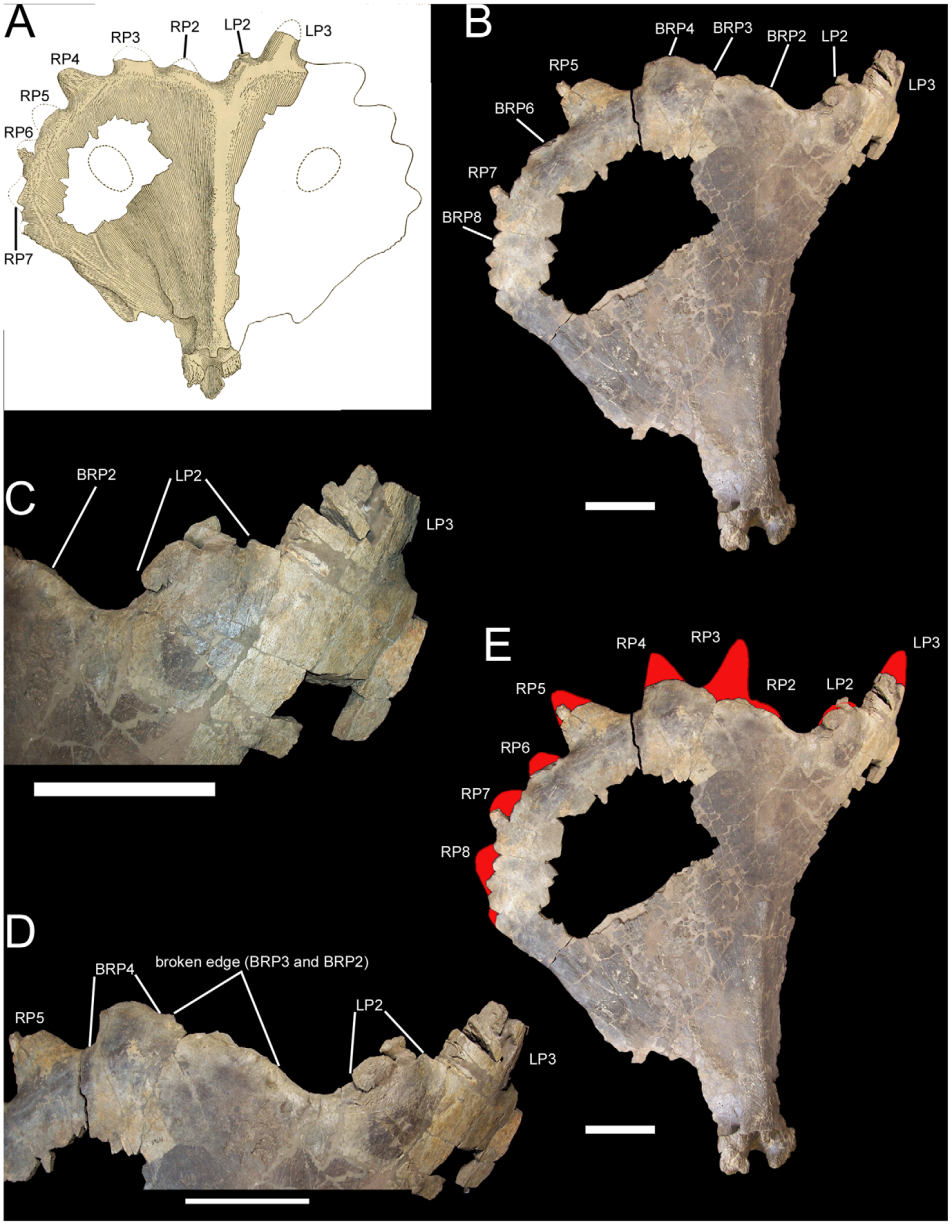
USNM 14765, parietal of *Rubeosaurus ovatus*. (A) Original reconstruction of parietal in dorsal view (modified from Gilmore [18]). (B) Parietal as preserved in dorsal view, with revised interpretation of the right parietal processes. (C) Left P2 and P3 parietal processes in dorsal view, showing breakage and displacement of fragments of the left P2. (D) Caudal parietal bar in dorsal view, showing extent of the broken edge along its caudal margin, which, in the revised interpretation of the parietal processes, represents the bases of the right P2 and P3 processes. (E) Parietal in dorsal view, with new reconstruction of the parietal processes. *Abbreviations*: *BRP2*, base of right P2 process; *BRP3*, base of right P3 process; *BRP4*, base of right P4 process; *BRP6*, base of right P6 process; *BRP8*, base of right P8 process; *LP2*, left P2 process; *LP3*, left P3 process; *RP2*, right P2 process; *RP3*, right P3 process; *RP4*, right P4 process; *RP5*, right P5 process; *RP6*, right P6 process; *RP7*, right P7 process; *RP8*, right P8 process. Scale bars in B–E equal 10 cm. B–E are Copyright Smithsonian Institution, all rights reserved.

The parietal of USNM 14765 is missing most of its left side (Fig. 10A, B). Gilmore's [18] reconstruction of the parietal included small elliptical parietal fenestrae (Fig. 10A), whereas Dodson [28] raised the possibility that parietal fenestrae were absent. The area rostral to the purported right parietal fenestra is not as intact as Gilmore's reconstruction would suggest; it is not a continuous expanse of bone, but rather a collection of numerous small fragments suspended in filling substance (Fig. 10B). Thus, the right parietal fenestra could have been larger than that in Gilmore's reconstruction, but ultimately the size and even the presence of parietal fenestrae must remain ambiguous.

The left parietal processes of USNM 14765 are a small caudomedially-directed medial-most process with a tapering nascent spike lateral to it on the caudal margin of the parietal (Fig. 10B, C). The locations and morphologies of these two processes on the parietal of USNM 14765 closely resemble those of the P2 and P3 processes of subadult and adult *Styracosaurus albertensis* [11], *Einiosaurus procurvicornis* (Fig. 7A, B, D, E), and *Achelousaurus horneri* (Fig. 8A, B). Therefore, these two processes on USNM 14765 are interpreted as the left P2 and P3. The left P2 has suffered damage and consists of two obviously displaced fragments, so that this process probably appears more prominent than it actually was (Fig. 10C).

A fresh examination of USNM 14765 led the author to reconsider the processes on the right side of the parietal. The original reconstruction of the parietal processes by Gilmore [18] included six processes (P2–P7) on the right side of the parietal (Fig. 10A). However, the bone surface between the right P2 and P3 of Gilmore's reconstruction is actually a broken edge (Fig. 10D); thus, the extent of the broken edge along the caudal margin of the caudal parietal bar is greater than is shown in Gilmore's reconstruction. Furthermore, immediately lateral to this broken edge is a small section of unbroken bone that curves caudally as if forming the base of a parietal process (Fig. 10D). The continuous broken edge along the right half of the caudal parietal bar is considerably wider mediolaterally than the base of the left P2 process, suggesting that the broken edge actually represents the broken bases of two parietal processes rather than one (the right P2) as in Gilmore's reconstruction. The broken edge is herein interpreted as corresponding to the bases of the right tab-like P2 and immediately adjacent P3 spike; the bases of the P2 and P3 processes are closely adjacent on the left side of the parietal of USNM 14765 (Fig. 10B, C) and on both sides of the parietal of USNM 11869, the holotype of *Rubeosaurus ovatus* (Fig. 1). This reinterpretation of the right parietal processes of USNM 14765 means that the nascent spike preserved on the right lateral parietal bar is not the right P4 as in Gilmore's reconstruction, but rather the right P5 (Fig. 10B); this process is missing its tip, and so would have been even longer than what is preserved on USNM 14765. The presence of a P5 spike on the parietal of USNM 14765 has important ramifications for the affinities of the specimen.

Incipient P5 spikes are also present on the parietals of subadult *Styracosaurus* (figs. 9A, 13A, 14A in [11]), but USNM 14765 differs from those specimens in lacking P1 processes on the dorsal surface of the parietal and thus should not be referred to *Styracosaurus*. USNM 14765 is also stratigraphically separated from specimens of *Styracosaurus* from the upper Dinosaur Park Formation of Alberta [11], but came from approximately the same level as the holotype of *Rubeosaurus* in the Upper Two Medicine Formation (see “Locality and Horizon” above). Among centrosaurines from the Upper Two Medicine Formation, only USNM 11869, the holotype of *Rubeosaurus*, exhibits an unequivocal P5 spike. Specimens of *Achelousaurus* only have spikes at the P3 positions (Fig. 8). Some specimens of *Einiosaurus* bear a spike at the P4 position that is shorter than the P3 spike but larger than the adjacent P5 epiparietal (Fig. 7C–E), but none in which the lateral parietal bar is preserved display a P5 spike (Fig. 7A, C, E). Therefore, USNM 14765 may be referred to *Rubeosaurus ovatus*. The right P6 and P8 of USNM 14765 are broken at their bases, while approximately the rostral third of the right P7 is preserved (Fig. 10B). The new interpretation of the parietal prompted a novel reconstruction of the parietal processes of USNM 14765 (Fig. 10E). It must be noted that the reconstructed left P3 of USNM 14765 appears to project caudolaterally (Fig. 10E), different from the caudomedially inclined P3 processes of USNM 11869, the holotype of *Rubeosaurus ovatus* (Fig. 1); given that the ontogeny of *Rubeosaurus* is almost totally unknown, it is impossible to say whether the inclination of the P3 spikes changed during development or whether the caudolateral inclination of the left P3 of USNM 14765 could be related to postmortem damage.

**Figure 11:**
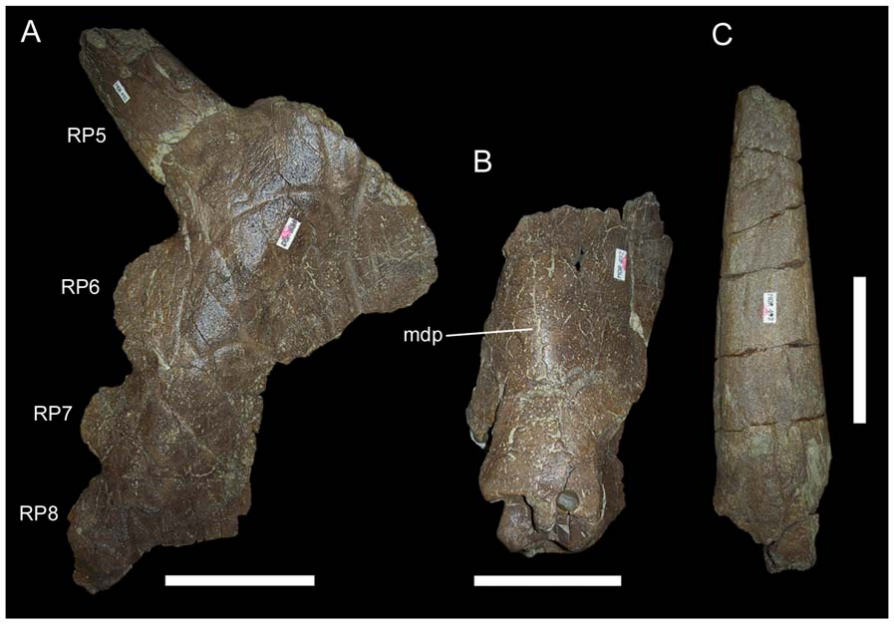
MOR 492, incomplete parietal of *Rubeosaurus ovatus*. (A) right lateral parietal bar in dorsal view; (B) partial median parietal bar in dorsal view; (C) P3 or P4 spike in dorsal view. *Abbreviations*: *mdp*, median prominence; *RP5*, right P5 process; *RP6*, right P6 process; *RP7*, right P7 process; *RP8*, right P8 process. Scale bars equal 10 cm.

In addition to USNM 14765, another specimen, MOR 492, may be referred to *Rubeosaurus ovatus* [13]. MOR 492 represents either a late subadult or adult individual, as indicated by the presence of only rugose bone texture [29]–[31] on the median and right lateral parietal bars. MOR 492 exhibits an elongate recurved nasal horncore (Fig. 4) and a low, rounded postorbital horncore (Fig. 9B). Preserved portions of the parietal include a partial median bar with a midline prominence, right lateral bar with a broken spike that fits onto its base, and a longer tapering spike broken distal to its base (Fig. 11). McDonald and Horner [13] interpreted the shorter of the two spikes as the right P4 process and the longer spike as a medially-inclined P3 process, and suggested that differences between MOR 492 and USNM 11869 (relatively short P4 spike and lack of a P5 spike in MOR 492) might indicate polymorphisms in the parietal processes of *Rubeosaurus ovatus*, similar to those reported for processes P6 and P7 in *Styracosaurus albertensis* [11]. However, recent comparison of the right lateral parietal bar of MOR 492 to parietals of *Einiosaurus* and to USNM 11869 suggests that the reconstructed orientation presented by McDonald and Horner (fig. 6B in [13]) is not the most likely interpretation. Instead, the shape of the right lateral parietal bar of MOR 492 appears to fit better with a revised interpretation of the shorter spike as the P5, and the progressively more rostral epiparietals as P6, P7, and P8 (Fig. 11A). The loose elongate spike could pertain to either the P4 or the P3 position; its orientation (i.e. caudomedially or caudolaterally inclined) is therefore unknown. The right lateral parietal bar of MOR 492 is difficult to interpret and future investigations could favor the original interpretation of McDonald and Horner [13], the new interpretation propounded herein, or neither. As with USNM 14765, the presence of a P5 spike combined with stratigraphic congruence with USNM 11869 indicates that MOR 492 should be referred to *Rubeosaurus ovatus*. The revised interpretation of the parietal of MOR 492 removes evidence for polymorphisms in the parietal processes of *Rubeosaurus*; this is not to say that such variation in the parietal spikes of *Rubeosaurus* is impossible, only that MOR 492 should not be used as evidence for it.

#### Phylogenetic Analysis

USNM 14765 includes the most complete skull of *Rubeosaurus ovatus* yet known and presents cranial elements that are either unknown or incomplete in USNM 11869 and MOR 492. To investigate whether new data from USNM 14765 would refine the phylogenetic affinities of *Rubeosaurus*, the specimen was incorporated into the phylogenetic analysis of Farke et al. [6], the most comprehensive analysis of centrosaurine relationships available. The revised codings for *Rubeosaurus* were based upon USNM 11869, USNM 14765, and MOR 492.

**Figure 12:**
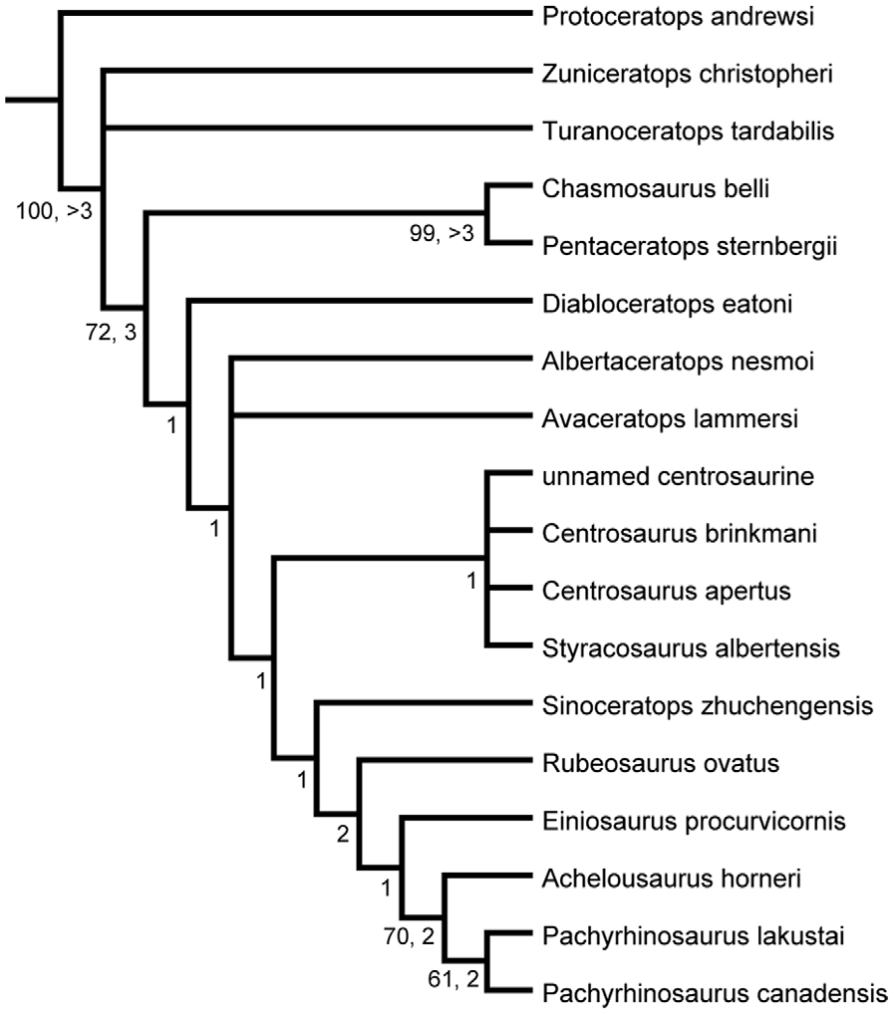
Phylogenetic relationships of *Rubeosaurus ovatus*. Strict consensus of three most parsimonious trees resulting from analysis of a data matrix modified from Farke et al. [6]. Numbers 1–3 to the left of and below nodes are Bremer support values. Numbers 61–100 to the left of and below some nodes are bootstrap percentages.

The analysis resulted in three most parsimonious trees of 130 steps each, with CI = 0.746 and RI = 0.772; the strict consensus tree is shown in Figure 12. Bremer support values and bootstrap percentages are generally low (Fig. 12). *Diabloceratops eatoni*, *Albertaceratops nesmoi*, and *Avaceratops lammersi* appear as basal centrosaurines. An unresolved clade of derived centrosaurines consists of taxa from the middle Campanian of Alberta, including the unnamed new centrosaurine, *Centrosaurus brinkmani*, *Centrosaurus apertus*, and *Styracosaurus albertensis*, all of which exhibit P1 parietal processes. *Rubeosaurus ovatus* is the sister taxon of a clade composed of *Einiosaurus procurvicornis*, *Achelousaurus horneri*, *Pachyrhinosaurus lakustai*, and *Pachyrhinosaurus canadensis* (Fig. 12). This position is supported by two synapomorphies: 59^0^ (shape of P2, low D-shaped process, wider than long [6]) and 61^1^ (shape of P3, elongate flattened process or spike [6]). Although the results of this analysis support the generic distinction of *Rubeosaurus ovatus* from *Styracosaurus albertensis*, a detailed assessment of centrosaurine phylogeny and paleobiogeography would be premature in the face of new basal taxa still to be published [32], [33].

### 2. Indeterminate Two Medicine ceratopsids

#### USNM 12745

**Figure 13:**
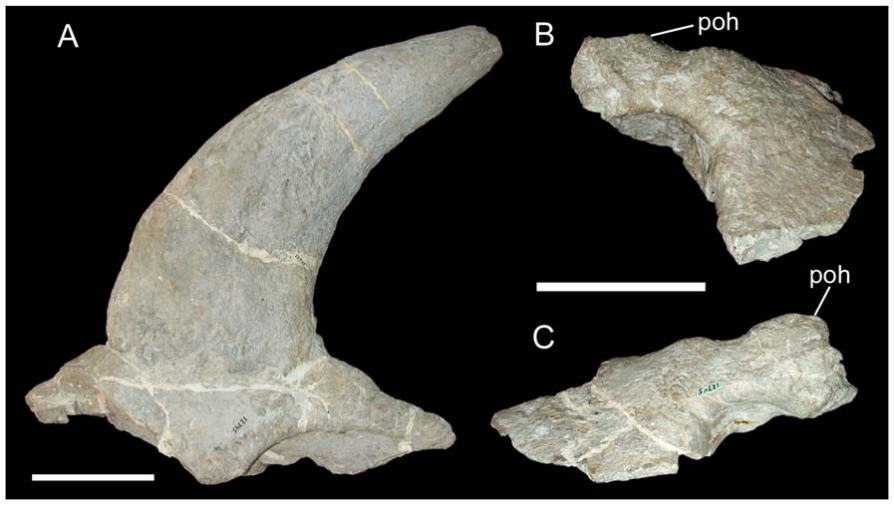
USNM 12745, indeterminate centrosaurine. Fused nasals in (A) right lateral view. Left (B) and right (C) postorbitals in lateral view. *Abbreviations*: *poh*, postorbital horncore. Scale bars equal 10 cm. Copyright Smithsonian Institution, all rights reserved.

USNM 12745 was discovered during Gilmore's 1928 expedition to the Landslide Butte area, the same expedition that produced USNM 11869, the holotype of *Rubeosaurus ovatus*. Gilmore [18] alluded to, but did not describe, this specimen and considered it referable to “*Monoclonius flexus*” ( = *Centrosaurus apertus* [9], [28]). In addition to several appendicular elements and vertebrae, USNM 12745 also includes partial fused left and right nasals and the left and right postorbitals. The nasals bear a large and complete horncore that curves rostrally towards its tip (Fig. 13A). This rostral curvature suggests that USNM 12745 might pertain to a subadult *Einiosaurus procurvicornis* that died before it could develop the strongly procurving nasal horncore characteristic of adults (Fig. 5D–F) [14]. However, in the absence of diagnostic parietal ornamentation, this possibility cannot be verified and USNM 12745 is best considered indeterminate. The postorbitals of USNM 12745 exhibit low, rounded, dorsally-directed horncores (Fig. 13B, C); the left postorbital horncore is missing its apex.

#### USNM 16512

**Figure 14:**
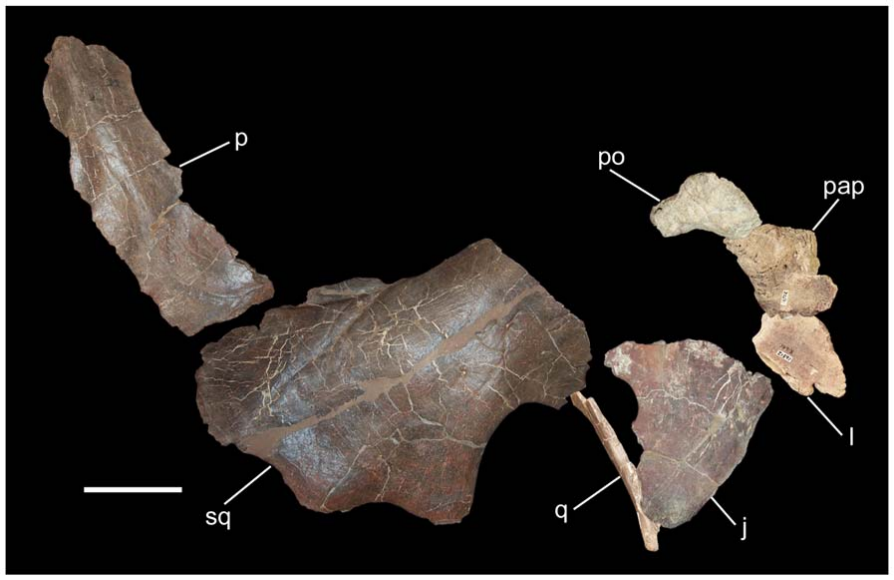
USNM 16512, indeterminate centrosaurine. Skull elements from the right side (except for the left postorbital and left quadrate, which have been reversed for the purpose of this figure) arranged in an approximation of life position. *Abbreviations*: *j*, jugal; *l*, lacrimal; *p*, lateral parietal bar; *pap*, palpebral; *po*, postorbital; *q*, quadrate; *sq*, squamosal. Scale bar equals 10 cm. Copyright Smithsonian Institution, all rights reserved.

USNM 16512 is a partial disarticulated centrosaurine skull collected during Gilmore's 1935 expedition to the Upper Two Medicine in the Landslide Butte area. The preserved elements include the left and right lacrimals, right palpebral, left and right jugals, partial left quadrate, partial left postorbital, left and right squamosals, and the right lateral parietal bar (Fig. 14). The left postorbital bears a short, rounded, dorsally-directed horncore like those of subadult and unmodified adult *Styracosaurus*, *Rubeosaurus*, and *Einiosaurus* (Fig. 9) [11], [13], [14]. The squamosals are of typical centrosaurine construction, with a distinct step in the contact with the parietal; the left squamosal bears four episquamosals, while the right has three. The right lateral bar is the only portion of the parietal present; it bears four epiparietals. The lack of the caudal parietal bar and nasals renders firm identification of USNM 16512 impossible. The presence of only adult bone texture on the right lateral parietal bar does indicate that the specimen is probably from either a late subadult or an adult individual, as this condition occurs in stages G, H, and I (adult) of Brown et al. [31].

#### MOR 449

**Figure 15:**
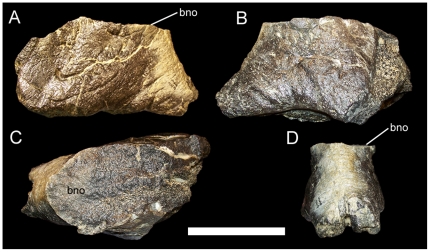
MOR 449, indeterminate ceratopsid. Fused left and right nasals in (A) right lateral, (B) left lateral, (C) dorsal, and (D) rostral views. *Abbreviations*: *bno*, base of nasal ornamentation. Scale bar equals 10 cm.

MOR 449 consists of partial fused left and right ceratopsid nasals, found near Landslide Butte at approximately the same stratigraphic level as specimens of *Rubeosaurus* and the type quarry of *Brachyceratops* [13]. The dorsal surface of the nasals bears the broken base of some manner of nasal ornamentation (Fig. 15). The seemingly wider base of this ornamentation compared to its length was used by McDonald and Horner [13] to argue for the possible presence of a second centrosaurine taxon 60 meters below the Bearpaw Formation, with a form of nasal ornamentation distinct from the laterally compressed horncore of MOR 492 (*Rubeosaurus*). However, the nasal horncore of MOR 492 has suffered some crushing and cracking (Fig. 4) that might make it appear more laterally compressed compared to MOR 449 than it actually was. Therefore, it is entirely possible that the broken base on MOR 449 supported an elongate, erect nasal horncore like that of MOR 492 and that MOR 449 could be referable to *Rubeosaurus ovatus*. However, in the absence of the rest of the nasal ornamentation, it is most prudent to simply regard MOR 449 as an indeterminate ceratopsid and to conclude that it does not indicate the presence of a second centrosaurine 60 meters below the top of the Two Medicine.

#### MOR 464

**Figure 16:**
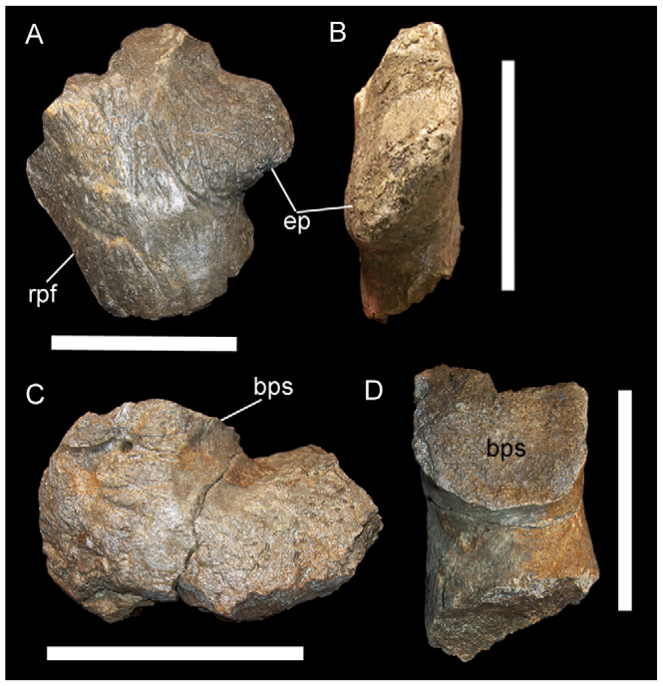
MOR 464, indeterminate centrosaurine. Fragment of lateral parietal bar in (A) dorsal and (B) lateral views. Fragment of caudal parietal bar in (C) dorsal and (D) caudal views. *Abbreviations*: *bps*, base of parietal spike; *ep*, epiparietal; *rpf*, rim of parietal fenestra. Scale bars equal 10 cm.

MOR 464 is a fragmentary skull from the vicinity of Landslide Butte that includes the basioccipital and several pieces of the parietal. A fragment of the left or right lateral parietal bar bears an epiparietal with its long axis oriented at an angle to the lateral margin of the parietal (Fig. 16A, B); this imbrication effect is present in adult centrosaurines [29] and indicates that MOR 464 represents such an animal. A different parietal fragment exhibits a raised broken surface that probably corresponds to the base of a parietal spike (Fig. 16C, D); however, because all three diagnostic centrosaurines from the Upper Two Medicine (*Rubeosaurus*, *Einiosaurus*, and *Achelousaurus*) exhibit at least one spike on either side of the parietal, this feature does not elucidate the affinities of MOR 464. Thus, MOR 464 is best considered an indeterminate centrosaurine.

## Methods

The phylogenetic analysis utilized the matrix and character list of Farke et al. [6]. In addition to further codings for *Rubeosaurus*, changes were made to the codings of several other taxa (S1, centrosaurine data matrix). Also, to format the matrix for use with TNT [34], hyphens (-) were replaced with question marks (?). Finally, two characters, 26 and 27 of Farke et al. [6], were slightly modified (S2, characters modified from Farke et al.). Otherwise, the data matrix and characters of Farke et al. [6] remained unchanged. The matrix was analyzed in TNT using a traditional search. The tree bisection-reconnection algorithm was used with Wagner starting trees, a random seed of one, and 10,000 replicates with 10 trees saved per replication. Character 20 was ordered (additive in the terminology of TNT), as in Farke et al. [6]. Bremer and bootstrap support were calculated in TNT; a standard bootstrap (sample with replacement in the terminology of TNT) calculation was carried out using a traditional search with 10,000 replicates and instruction to collapse groups with bootstrap percentages less than 50%.

## Supporting Information

Table S1 **Data matrix used in the phylogenetic analysis of Centrosaurinae.**
(XLS)

Table S2 **Changes made to the character list of Farke et al.** [**6**] **.**
(DOC)
