# Supplementary material for: A Subadult Specimen of Rubeosaurus ovatus (Dinosauria: Ceratopsidae), with Observations on Other Ceratopsids from the Two Medicine Formation
Source: PLoS One. 2011 Aug 10;6(8):e22710. doi: 10.1371/journal.pone.0022710 (PMC3154267; doi:10.1371/journal.pone.0022710)
Supplement: Table S2 — Changes made to the character list of Farke et al. [6] . (DOC) [file pone.0022710.s002.doc]

**S2, Changes Made to the Character List of Farke et al. (in press).**

Changes are highlighted in red.

26. Postorbital, position of supraorbital ornamentation: (0) centered rostrodorsal or dorsal to orbit, narrow base with caudal margin of supraorbital horncore extending to or only slightly behind caudal margin of orbit; (1) centered caudodorsal to orbit, broad base with caudal margin of supraorbital horncore extending well behind caudal orbit (modified from Lehman 1996, character 9).

27. Postorbital, orientation of supraorbital ornamentation base: (0) dorsally directed; (1) dorsolaterally directed (modified from Sampson et al. 2010, character 38).

Lehman, T. M. 1996. A horned dinosaur from the El Picacho Formation of west Texas, and review of ceratopsian dinosaurs from the American Southwest. Journal of Paleontology 70: 494-508.

Sampson, S. D., Loewen, M. A., Farke, A. A., Roberts, E. M., Forster, C. A., Smith, J. A., and

Titus, A. L. 2010. New horned dinosaurs from Utah provide evidence for intracontinental dinosaur endemism. *PLoS ONE* 5(9): e12292. doi:10.1371/journal.pone.0012292.
